# Eryptosis in renal anemia: mechanisms, clinical implications, and therapeutic targeting

**DOI:** 10.3389/fphar.2025.1718803

**Published:** 2025-11-26

**Authors:** Xiang Deng, Xiangyu Gong, Yi Huang, Jie Zhou, Sichong Ren

**Affiliations:** 1 Department of Nephrology, School of Clinical Medicine and The First Affiliated Hospital of Chengdu Medical College, Chengdu, China; 2 School of Clinical Medicine, Chengdu Medical College, Chengdu, China; 3 TCM Preventative Treatment Research Center of Chengdu Medical College, Chengdu, China; 4 The First Affiliated Hospital of Traditional Chinese Medicine of Chengdu Medical College·Xindu Hospital of Traditional Chinese Medicine, Chengdu, China

**Keywords:** eryptosis, renal anemia, uremic toxin, CKD, EPO

## Abstract

Renal anemia is one of the most common complications of chronic kidney disease (CKD) and is associated with serious clinical consequences. Its prevalence increases significantly as renal function declines, affecting over 90% of dialysis patients. Traditionally, the pathophysiology of renal anemia has been centered on two primary mechanisms: absolute or relative deficiency of erythropoietin (EPO) and disorders of iron metabolism. However, even with treatment using erythropoiesis-stimulating agents (ESAs) and iron supplements, approximately half of the patients exhibit a hypo-responsive or suboptimal correction of anemia, suggesting the involvement of other significant mechanisms in the development and progression of renal anemia. In recent years, eryptosis, a novel mechanism, has garnered increasing attention. Eryptosis is a form of programmed cell death specific to erythrocytes, sharing similarities with but distinct from apoptosis in nucleated cells. It is characterized by cell shrinkage, membrane blebbing, and phosphatidylserine (PS) externalization. In the CKD milieu, various uremic toxins, oxidative stress, and inflammatory factors can accelerate the eryptosis process, leading to a significant reduction in red blood cell lifespan—from the normal 120 days toonly 60–90 days in CKD patients. This accelerated eryptosis represents a major contributing factor to renal anemia. This review aims to systematically summarize the association between renal anemia and eryptosis, providing an in-depth exploration of its molecular mechanisms, clinical implications, and therapeutic potential. Distinguishing itself from existing reviews, this article will focus on the central role of eryptosis in renal anemia. It integrates the latest evidence from basic research and clinical data to propose innovative therapeutic strategies targeting eryptosis, thereby offering new perspectives for improving the current management of renal anemia.

## 1 Introduction

CKD, which is defined by the gradual loss of kidney function and progressive structural damage, has become a pressing public health concern globally, including in China. Current estimates indicate that CKD affects approximately 11%–13% of the worldwide population. Among them, renal anemia is one of the most common and important complications of CKD. As defined in the clinical practice guidelines established by Kidney Disease: Improving Global Outcomes (KDIGO), the diagnostic threshold for anemia in CKD is a hemoglobin (Hb) level below 13.0 g/dL for male patients and under 12.0 g/dL for females who are not pregnant (KDIGO, 2024). Its pathogenesis is primarily attributed to reduced synthesis of EPO by the diseased kidneys and/or disruptions in iron metabolism (Bonom et al., 2016; Babitt and Lin, 2012). Relative EPO deficiency is further evidenced by studies showing blunted EPO responses to anemia in CKD patients, where serum EPO levels fail to rise adequately in proportion to hemoglobin decline (Artunc and Risler, 2007). Contemporary management strategies encompass the administration of oral or intravenous iron supplements, erythropoiesis-stimulating agents (ESAs), and red blood cell transfusions. The 1980s witnessed a landmark advancement in managing renal anemia with the introduction of erythropoiesis-stimulating agents (ESAs). This innovation significantly enhanced the quality of life for end-stage renal disease (ESRD) patients and reduced reliance on blood transfusions (Schmid and Schiffl, 2010). Furthermore, ESA therapy has been shown to decrease dialysis frequency and alleviate demands on healthcare resources, particularly among chronic kidney disease (CKD) patients dependent on dialysis. However, subsequent investigations revealed that nearly 10% of individuals treated with ESAs develop resistance to erythropoietin, potentially associated with iron insufficiency and inflammatory conditions (Macdougall, 2024). Many studies have revealed that escalating ESA dosages is correlated with elevated risks of malignancy, all-cause mortality, and increased hospital admissions. More recently, hypoxia-inducible factor prolyl hydroxylase inhibitors (HIF-PHIs) have received clinical approval for their ability to promote hematopoiesis through enhancing endogenous EPO production (McCallum et al., 2022). Their application, however, necessitates thorough assessment of oncological histories prior to initiation. Several challenges persist in renal anemia management, such as establishing personalized target hemoglobin concentrations, minimizing dose-dependent adverse events, and mitigating economic constraints on patients. The primary contributors to renal anemia involve deficient EPO synthesis and inadequate iron availability for erythrocyte production. Research indicates that the average red blood cell lifespan (RBCL) progressively shortens across CKD stages 1–5, reported as 122 ± 50, 112 ± 26, 90 ± 32, 88 ± 28, and 60 ± 24 days, respectively (Li et al., 2019). During early CKD stages, rising EPO levels constitute a compensatory mechanism addressing hemoglobin decline from erythrocyte degradation. However, the capacity for new RBC production fails to counterbalance these losses promptly, potentially worsening anemia, notably in uremic cases (Hamza et al., 2020). Among dialysis-dependent ESRD patients, eryptosis—accelerated programmed erythrocyte death—further compounds the reduction of circulating RBCs (Abed et al., 2014). Although ESAs offer substantial benefits, they do not represent a comprehensive solution for renal anemia. Emerging insights into the mechanisms and modifiers of eryptosis hold potential for refining therapeutic strategies. This review summarizes the pathophysiology of eryptosis and its key determinants based on contemporary scientific evidence.

## 2 Molecular mechanism and regulatory network of eryptosis

### 2.1 Molecular mechanism of eryptosis

**FIGURE 1:**
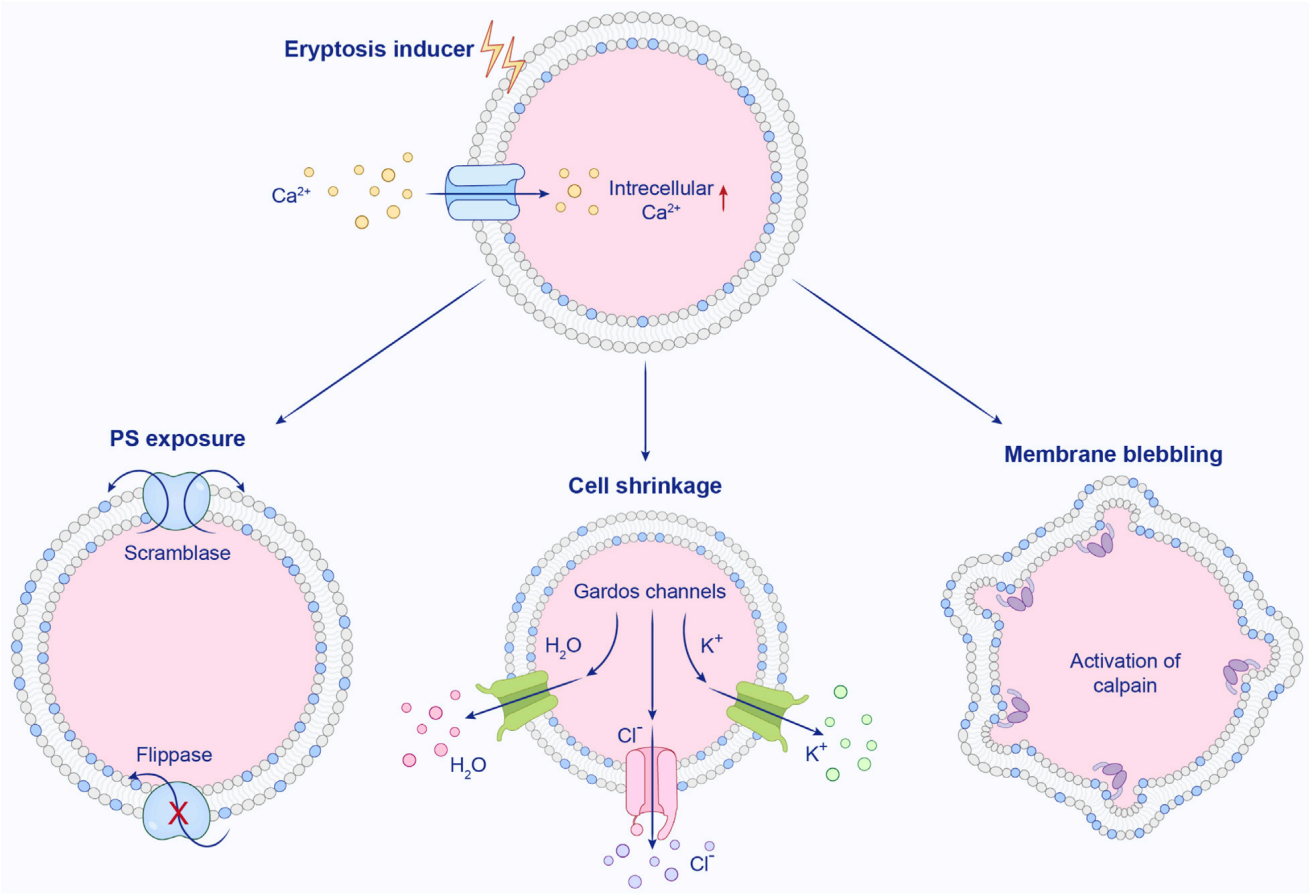
A central pathway in eryptosis is initiated by the elevation of cytosolic Ca^2+^, resulting from the opening of Ca^2+^-permeable cation channels that allow extracellular Ca^2+^ to enter the erythrocyte. (1) The rise in intracellular Ca^2+^ subsequently stimulates scramblases while inhibiting flippases, leading to the externalization of phosphatidylserine (PS) on the outer membrane. (2) High Ca^2+^ levels trigger the activation of Ca^2+^-sensitive potassium channels, known as Gardos channels. This activation promotes the efflux of KCl and water, resulting in cellular shrinkage. (3) Calpain, a Ca^2+^-dependent cysteine endopeptidase, becomes activated and proteolytically cleaves cytoskeletal components, ultimately inducing membrane blebbing. Redrawn from Alghareeb et al. (2023), licensed under CC BY.

Eryptosis, the programmed death of erythrocytes, can be triggered prematurely under various pathological conditions or by therapeutic interventions, including oxidative stress, hyperosmolarity, heavy metal exposure, energy deficiency, xenobiotics, and antibiotics (Ghashghaeinia et al., 2012; Lang K. S. et al., 2005). As erythrocytes circulate, they encounter varying mechanical pressures and chemical environments. They may experience oxidative stress in pulmonary capillaries or osmotic shock in the renal vasculature. As illustrated in Figure 1, characteristic alterations in the erythrocyte membrane, such as blebbing, cellular shrinkage, and externalization of phosphatidylserine (PS), are observed during eryptosis, mirroring features typical of apoptotic cell death (Berg et al., 2001).

A key mediator of eryptosis is the rise in cytosolic Ca^2+^, which can be induced by osmotic shock, oxidative stress, or other stimuli (Qadri et al., 2012). Although erythrocytes lack organelles, Ca^2+^ influx occurs through several Ca^2+^-permeable channels. These include TRPC channels (e.g., TRPC6 in humans (Foller et al., 2008)), ionotropic glutamate receptors (NMDA (Makhro et al., 2013; Kaestner et al., 2020) and AMPA receptors (Föller et al., 2009a)), the mechanosensitive PIEZO1 channel (Cahalan et al., 2015), and voltage-gated Cav2.1 channels (Andrews et al., 2002). The activation of these channels is facilitated by conditions such as high ROS, hyperosmolarity, or chloride depletion (Duranton et al., 2002). Prostaglandin E2 (PGE2) also promotes Ca^2+^ influx and membrane vesiculation (Qadri et al., 2012). Elevated intracellular Ca^2+^ activates Ca^2+^-sensitive Gardos channels (KCa3.1, encoded by KCNN4), resulting in K^+^ efflux, membrane hyperpolarization, Cl^−^ loss, and subsequent osmotic cell shrinkage (Qadri et al., 2012; Föller and Lang, 2020; Rapetti-Mauss et al., 2017). Concurrently, Ca^2+^ inhibits flippase and activates scramblase, disrupting phospholipid asymmetry and causing PS exposure on the outer membrane leaflet (Rapetti-Mauss et al., 2017; Pretorius et al., 2016). This surface PS enables macrophage recognition via PS receptors, leading to phagocytosis and clearance of eryptotic cells (Boas et al., 1998; Nicolay et al., 2006). Ca^2+^ also activates the cysteine protease calpain-1, which degrades cytoskeletal proteins such as the ankyrin-R complex, promoting membrane blebbing and increasing cellular adhesiveness (Restivo et al., 2021). Although calpain-1 is implicated in cytoskeletal breakdown, its genetic ablation does not alter erythrocyte lifespan, indicating possible compensatory mechanisms (Wieschhaus et al., 2012). Additional pathways contribute to eryptosis independently or synergistically with Ca^2+^. Sphingomyelinase (endogenous or exogenous) cleaves sphingomyelin to generate ceramide, which facilitates PS externalization and enhances Gardos channel activity (Lang K. S. et al., 2005). Ceramide also promotes inflammatory responses. Under osmotic stress, phospholipase activation stimulates platelet-activating factor (PAF) production, further supporting membrane scrambling (Lang K. S. et al., 2005). Energy deficiency also exacerbates eryptosis through specific signaling pathways. Janus-activated kinase 3 (JAK3) phosphorylates the regulatory tyrosine site Tyr 980, contributing to membrane scrambling under conditions of energy deprivation (Li et al., 2019; Lang et al., 2015b). Similarly, casein kinase 1α (CK1α) has been implicated in the elevation of intracellular Ca^2+^ and promotion of eryptosis following energy loss or oxidative stress. Pharmacologic activation of CK1 opens cation channels, permitting Ca^2+^ influx into the erythrocyte (Lang F. et al., 2012; Lang and Lang, 2015). Overall, eryptosis is a multi-factorial process driven by Ca^2+^ and other signaling molecules, resulting in cell shrinkage, PS exposure, and ultimately phagocytic clearance, which may play a significant role in various anemias including renal anemia.

### 2.2 Regulatory network of eryptosis

**FIGURE 2:**
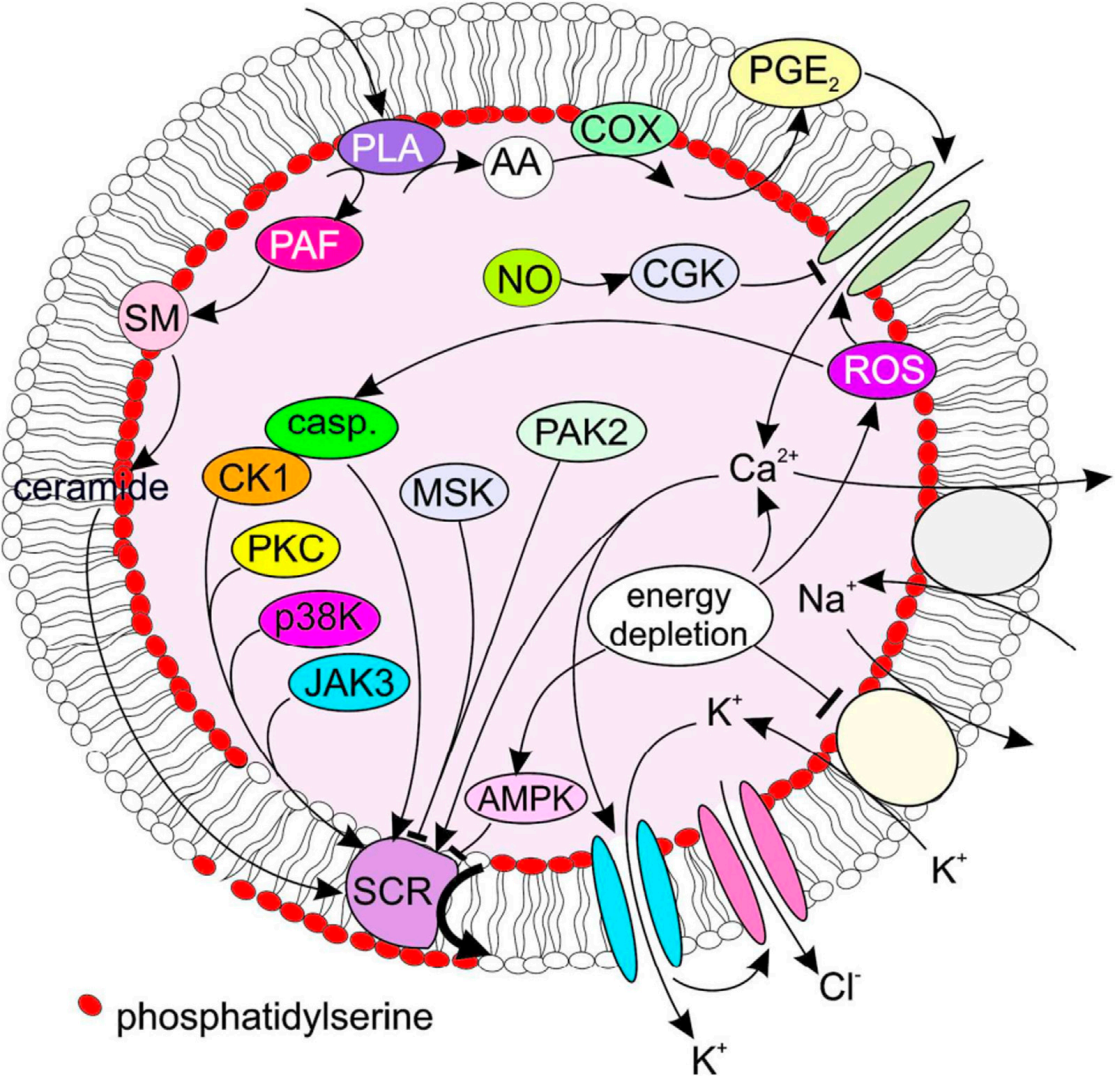
Key signaling mechanisms implicated in eryptosis. AA, arachidonic acid; AMPK, AMP-activated kinase; casp, caspases; cGK, cGMP-dependent protein kinase 1; CK1, casein kinase 1; COX, cycloxygenase; JAK3, janus kinase 3; MSK, mitogen- and stress- activated kinase; PAK2, p21-activated kinase 2; PAF, platelet activating factor; PGE2, prostaglandin E2; PLA, phospholipase A; SCR, scramblase; SM, sphingomyelinase. Reproduced with permission from Lang E. et al. (2017), copyright 2017 UICC.

In addition to cytosolic Ca^2+^ elevation, ceramide serves as a principal trigger of eryptosis (Lang et al., 2004). Its biosynthesis is catalyzed by sphingomyelinase, a phosphodiesterase that hydrolyzes the membrane sphingolipid sphingomyelin (SM) to generate ceramide (Zeidan and Hannun, 2010). Unlike its established role in CD95-mediated apoptosis of nucleated cells, ceramide promotes eryptosis primarily by enhancing Ca^2+^ sensitivity rather than directly facilitating Ca^2+^ entry (Gulbins et al., 1995). Beyond ceramide and Ca^2+^, eryptosis is fine-tuned by a cohort of kinases that interface with multiple signaling nodes illustrated in Figure 2. These include AMP-activated protein kinase (AMPK), which modulates erythrocyte survival via cation flux regulation and crosstalk with p21-activated kinase 2 (PAK2); proteomic analyses of AMPK-deficient erythrocytes have identified PAK2 as a downstream effector, highlighting their cooperative role in restraining excessive eryptosis (Föller et al., 2009b; Zelenak et al., 2011). Cyclic GMP-dependent protein kinase type I (cGKI) exerts a cytoprotective function, as cGKI-deficient mice exhibit anemia and splenomegaly due to accelerated erythrocyte clearance—underscoring cGKI’s capacity to buffer pro-eryptotic signals (Föller et al., 2008a). Janus kinase3 (JAK3) is expressed in erythrocytes, phosphorylated upon energy depletion, and involved in regulating eryptosis by triggering cell membrane scrambling (Bhavsar et al., 2011). Protein kinase CK1α regulates eryptosis by modulating cytosolic Ca^2+^ activity, and its activator pyrvinium pamoate further influences this process via activating Cl^−^-sensitive Ca^2+^-permeable cation channels and inhibiting Ca^2+^-activated K^+^ channels (Kucherenko et al., 2012; Zelenak et al., 2012). Additional kinases, such as cyclin-dependent kinase 4 (CDK4) and mitogen- and stress-activated kinases 1/2 (MSK1/2), contribute non-redundant regulatory roles: pharmacological inhibition of CDK4 attenuates eryptosis, while deficiency in MSK1/2 accelerates erythrocyte apoptotic turnover *in vivo*, emphasizing their importance in maintaining erythrocyte homeostasis (Lang et al., 2015a; Lang et al., 2015b). In addition, lipid metabolism further acts as a critical regulatory node within complicated network. Phospholipase A (PLA)-mediated cleavage of membrane phospholipids releases arachidonic acid (AA), a substrate for cyclooxygenase (COX) in the biosynthesis of prostaglandin E_2_ (PGE_2_) (Lang P. A. et al., 2005). PGE_2_ not only enhances Ca^2+^ influx but also interacts with platelet-activating factor (PAF) to amplify scramblase (SCR)-mediated phosphatidylserine (PS) externalization under osmotic stress (Setty and Betal, 2008). Counterbalancing these pro-eryptotic lipid signals, nitric oxide (NO) inhibits eryptosis by suppressing cation channel activation and reducing PS externalization, thereby preventing premature erythrocyte clearance (Nicolay et al., 2008). Ion transport mechanisms, including the plasma membrane Ca^2+^ ATPase (PMCA) and anion exchanger 1 (AE1, Band 3), also function as checkpoints: PMCA extrudes excess Ca^2+^ to limit overload, with its activity modulated by ceramide or AA, while AE1 mutations disrupting ion balance promote eryptosis (Nicolay et al., 2008; Abbas et al., 2018). Collectively, these interconnected pathways—centered on ceramide, kinase signaling, lipid mediators, and ion transport—orchestrate the fine regulation of eryptosis as visualized in Figure 2.

## 3 The activation mechanism of eryptosis in renal anemia

**FIGURE 3:**
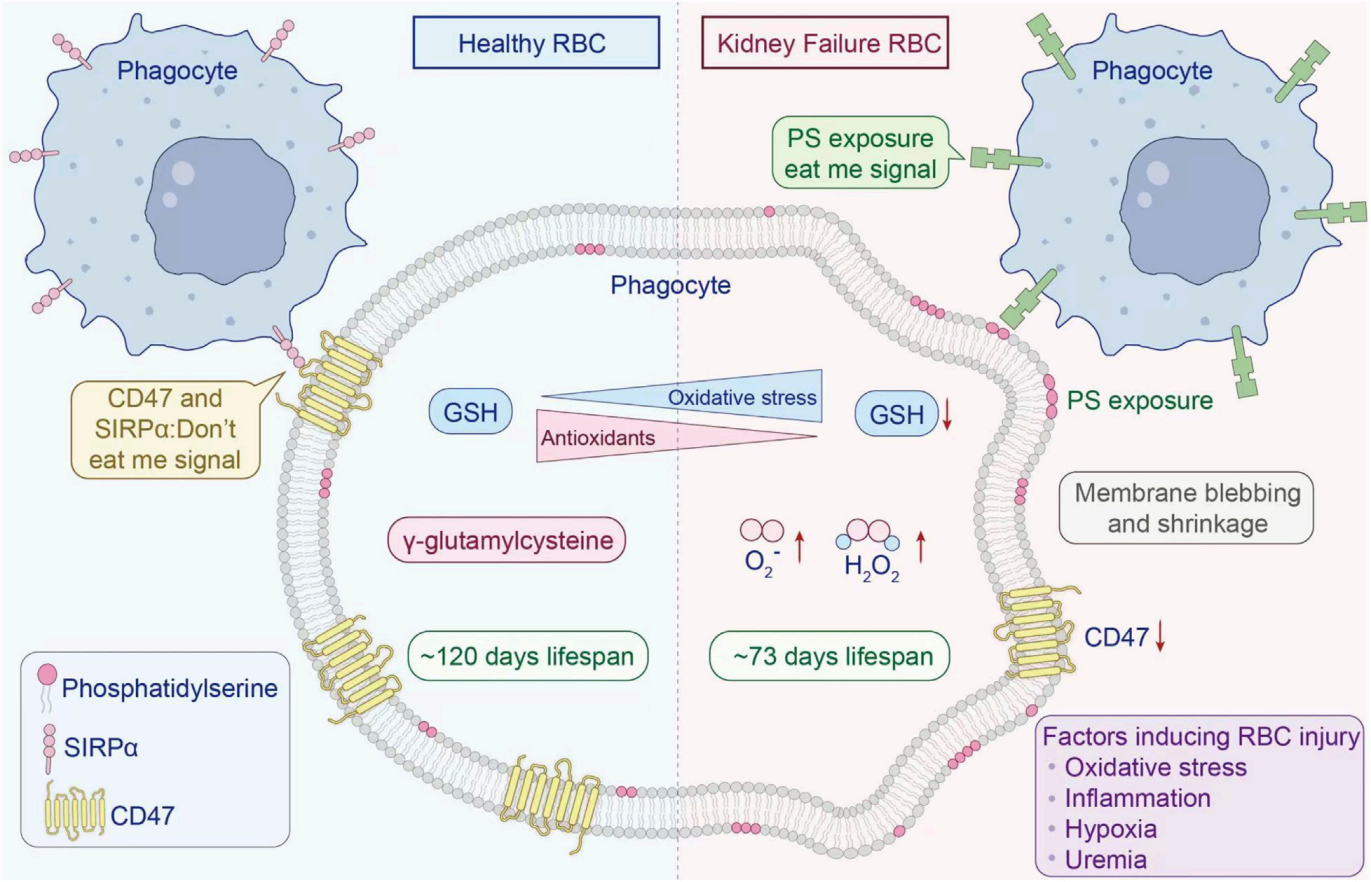
Under physiological conditions (left), the integral membrane protein CD47 binds to phagocytic cell receptors, delivering a “do not eat me“ signal to inhibit erythrophagocytosis. CD47 expression gradually decreases over ∼120 days, enabling senescent RBC clearance via the monocyte-macrophage system. In renal failure (right) and CKD, RBC lifespan is significantly shortened. Multiple pathological factors (uremia, hypoxia, inflammation, oxidative stress) induce cellular damage signals and trigger eryptosis. RBCs from CKD patients show elevated oxidant production, impaired antioxidant recycling, and enhanced PS externalization. Pro-inflammatory monocytes and tissue-resident macrophages recognize surface-exposed PS, accelerating RBC clearance from circulation. Redrawn from Dias et al. (2020), licensed under CC BY.

In the uremic milieu of CKD, a constellation of factors—including uremic toxin accumulation, chronic inflammation, oxidative stress, and hypoxia—converge to induce cellular damage signals that promote eryptosis, the programmed death of erythrocytes. This process is characterized by PS externalization, cell shrinkage, membrane blebbing, and ultimately, premature clearance of RBCs from circulation. As illustrated in Figure 3, these structural and biochemical alterations significantly shorten RBC lifespan, exacerbating anemia and contributing to the high cardiovascular risk observed in CKD patients. The following sections delve into the specific roles of uremic toxins, oxidative stress, inflammation, and iron imbalance in triggering eryptosis, providing a comprehensive overview of the pathophysiological mechanisms underlying renal anemia. Recent reviews highlight the physiological role of eryptosis in maintaining erythrocyte homeostasis by clearing damaged cells, but in CKD, this process becomes dysregulated, leading to excessive loss and anemia (Tkachenko et al., 2025).

### 3.1 The effect of uremic toxins

The progression of CKD is accompanied by the accumulation of diverse uremic toxins, which significantly accelerates the process of eryptosis. These toxins, originating primarily from endogenous metabolism and dietary protein breakdown, are key contributors to enhanced eryptosis in CKD. Uremic toxins are commonly classified into three categories: water-soluble toxins, protein-bound toxins, and intermediate molecular-weight substances (Vanholder et al., 2003). Binding to albumin can impede the removal of certain toxins through dialysis (Sirich et al., 2014). In end-stage renal disease (ESRD), the plasma environment plays a critical role in promoting eryptosis, as demonstrated by observations that healthy red blood cells (RBCs) incubated in plasma from ESRD patients exhibit markedly elevated PS externalization (Abed et al., 2014). The average RBC lifespan is significantly shorter in hemodialysis (HD) patients (89 ± 28 days) compared to healthy individuals (128 ± 28 days) (Sato et al., 2012). Multiple uremic toxins have been identified as inducers of eryptosis, including indoxyl sulfate (IS) (Ahmed et al., 2013a), phosphate (Voelkl et al., 2013), acrolein (Ahmed et al., 2013b), vanadate (Föller et al., 2008b), and indole-3-acetic acid (IAA) (Gao et al., 2015) as listed in Table 1.

**TABLE 1:** Key uremic toxins implicated in eryptosis in CKD.

Uremic toxin	Functional classification	Source	Mechanisms of eryptosis induction	Clinical relevance	References
Indoxyl sulfate (IS)	Protein-bound toxin	Tryptophan is metabolized to indole by gut microbiota, which is further oxidized and sulfated in the liver to form IS	Elevates intracellular Ca^2+^ and induces membrane phospholipid scrambling, leading to PS externalization; Increases ceramide levels; Triggers via glutathione (GSH)-dependent pathways; Activates p38 MAP kinase; Synergizes with reactive oxygen species (ROS)	Correlates with increased cardiovascular event incidence; Promotes RBC-derived MP generation, enhancing thrombosis and impairing microcirculation	Ahmed et al. (2013a), Meyer and Hostetter (2012), Wu et al. (2017), Takada et al. (2018), Lin et al. (2012), Dou et al. (2007), Barreto et al. (2009)
Indole-3-acetic acid (IAA)	Protein-bound toxin	Metabolism of tryptophan by gut microbiota	Promotes PS externalization and generation of RBC-derived MPs	Enhances thrombosis and contributes to cardiovascular risk in CKD	Ahmed et al. (2013a), Gao et al. (2015)
Phosphate	Water-soluble inorganic toxin	Dietary intake; cellular metabolism	Activates p38 MAP kinase upon systemic accumulation; Calcium phosphate crystals further stimulate p38 kinase activity and promote platelet-activating factor (PAF) production	Pathogenic in CKD due to impaired renal excretion; exacerbates eryptosis and cardiovascular risk	Voelkl et al. (2013), Erem and Razzaque (2018), Lang et al. (2012b), Vervloet et al. (2017)
Vanadate	Inorganic toxin	Environmental exposure; dietary sources	Inhibits ATP production, inducing energy deficiency in RBCs; Alters redox state of glyceraldehyde-3-phosphate dehydrogenase, impairing glycolysis and worsening energy depletion; Potentiates the eryptotic effects of intracellular Ca^2+^	Accumulates in CKD; accelerates eryptosis via energy metabolism disruption	Föller et al. (2008b), Benabe et al. (1987), Hosokawa and Yoshida (1993)
Acrolein	Reactive aldehyde	Endogenous metabolism; dietary sources	Stimulates ceramide generation, increasing erythrocyte sensitivity to cytosolic Ca^2+^	Induces eryptosis and contributes to RBC lifespan shortening in CKD	Ahmed et al. (2013b), Benabe et al. (1987)

#### 3.1.1 Indoxyl sulfate (IS)

Indoxyl sulfate (IS) is classified as a protein-bound uremic toxin, with its biosynthesis originating from tryptophan metabolism: gut microbiota first convert tryptophan into indole, which is then transported to the liver for oxidation and sulfation to form IS (Meyer and Hostetter, 2012). As a protein-bound toxin, IS tightly associates with plasma proteins, hindering its glomerular filtration. Elimination instead depends on organic anion transporters 1 and 3 (OAT1/3) located on renal tubular epithelial cells, which facilitate its uptake from blood into the tubules (Wu et al., 2017). Efflux into the urine is mediated by the transporter ABCG2 (Takada et al., 2018). Mechanistically, IS induces eryptosis through multiple interconnected pathways: it elevates intracellular Ca^2+^ concentrations, triggering membrane phospholipid scrambling and subsequent phosphatidylserine (PS) externalization; increases ceramide levels to amplify eryptotic processes; activates p38 mitogen-activated protein kinase (p38 MAPK)—a signaling molecule involved in apoptotic cascades; and acts via glutathione (GSH)-dependent mechanisms, with synergistic effects alongside reactive oxygen species (ROS) to exacerbate erythrocyte death Mechanistically, IS induces eryptosis through multiple interconnected pathways: it elevates intracellular Ca^2+^ concentrations, triggering membrane phospholipid scrambling and subsequent phosphatidylserine (PS) externalization; increases ceramide levels to amplify eryptotic processes; activates p38 mitogen-activated protein kinase (p38 MAPK)—a signaling molecule involved in apoptotic cascades; and acts via glutathione (GSH)-dependent mechanisms, with synergistic effects alongside reactive oxygen species (ROS) to exacerbate erythrocyte death (Ahmed et al., 2013a; Dias et al., 2018; Gatidis et al., 2011). Clinically, elevated IS levels correlate strongly with increased cardiovascular event incidence in patients with CKD, primarily mediated by inducing oxidative stress in endothelial cells (Lin et al., 2012; Dou et al., 2007). Additionally, IS promotes the generation of red blood cell (RBC)-derived microparticles (MPs); these vesicles expose PS and other membrane antigens, enabling the binding of factor Xa and prothrombinase complexes, which amplifies thrombin production, accelerates coagulation, impairs microcirculation, and further elevates cardiovascular risk (Gao et al., 2015; Barreto et al., 2009). Notably, conventional hemodialysis fails to efficiently clear IS due to its protein-binding property, resulting in persistently elevated concentrations in CKD patients compared to healthy individuals (Ahmed et al., 2013a; Dias et al., 2021).

#### 3.1.2 Indole-3-acetic acid (IAA)

Indole-3-acetic acid (IAA), another protein-bound toxin, is also a product of tryptophan metabolism by gut microbiota. Similar to IS, IAA promotes PS externalization and the generation of RBC-derived MPs (Gao et al., 2015). During eryptosis, PS externalization is accompanied by MP release; these vesicles display not only PS but also other membrane antigens. Factor Xa and prothrombinase complexes bind to PS on MPs and RBC surfaces, amplifying thrombin production and accelerating coagulation. These processes contribute to impaired microcirculation and elevated cardiovascular risk in CKD. Although phosphate is essential for cellular metabolism, its excess—particularly under conditions of impaired renal excretion—becomes pathogenic (Erem and Razzaque, 2018). IAA exacerbates renal anemia by shortening RBC lifespan and enhances prothrombotic activity, thereby worsening the clinical outcomes of CKD patients (Gao et al., 2015).

#### 3.1.3 Phosphate

Phosphate, a water-soluble inorganic uremic toxin, is primarily derived from dietary intake and cellular metabolism (Voelkl et al., 2013). Under physiological conditions, the kidneys efficiently excrete excess phosphate; however, in CKD, impaired renal function leads to systemic phosphate accumulation, driving its pathogenic effects (Lang E. et al., 2012). Mechanistically, accumulated phosphate activates p38 MAPK—a downstream effector of phospholipase A2 signaling—which in turn promotes eryptosis (Börsch-Haubold et al., 1997). Phosphate overload in CKD not only exacerbates eryptosis and shortens RBC lifespan but also contributes to vascular calcification and other cardiovascular complications, making phosphate control a critical component of CKD management (Barreto et al., 2019).

#### 3.1.4 Vanadate

Vanadate, an inorganic uremic toxin, is acquired through environmental exposure and dietary sources. In CKD, reduced renal excretion leads to vanadate accumulation, which accelerates eryptosis through multiple metabolic disruptions (Vervloet et al., 2017). Specifically, vanadate inhibits ATP production in erythrocytes, creating an energy-deficient state that predisposes to eryptosis. It also alters the redox state of glyceraldehyde-3-phosphate dehydrogenase—a key enzyme in glycolysis—thereby impairing glucose metabolism and further worsening energy depletion in RBCs (Benabe et al., 1987). Additionally, vanadate potentiates the eryptotic effects of intracellular Ca^2+^, amplifying Ca^2+^-dependent pathways such as PS externalization and cell shrinkage. Clinically, vanadate accumulation in CKD contributes to progressive renal anemia by shortening RBC lifespan, highlighting the need for strategies to reduce its levels in uremic patients.

#### 3.1.5 Acrolein

Acrolein, another uremic toxin, is known to exacerbate oxidative injury, resulting in elevated ceramide production that contributes to a rise in cytosolic Ca^2+^ levels and thereby promotes eryptosis (Ahmed et al., 2013b). More recently, in 2024, Kopera et al. proposed that acrolein plays a key role in disrupting erythrocyte membrane integrity, inducing alterations in membrane architecture, cytosolic proteins, and osmotic fragility. Their findings indicated that these structural modifications occur in a dose-dependent manner (Kopera et al., 2024).

In conclusion, the accumulation of specific uremic toxins in CKD—such as indoxyl sulfate, phosphate, vanadate, and acrolein—promotes eryptosis through distinct but convergent mechanisms involving Ca^2+^ dysregulation, oxidative stress, energy depletion, and kinase activation. This toxin-driven reduction in erythrocyte survival not only exacerbates renal anemia but also promotes a prothrombotic state.

### 3.2 The effect of oxidative stress

**FIGURE 4:**
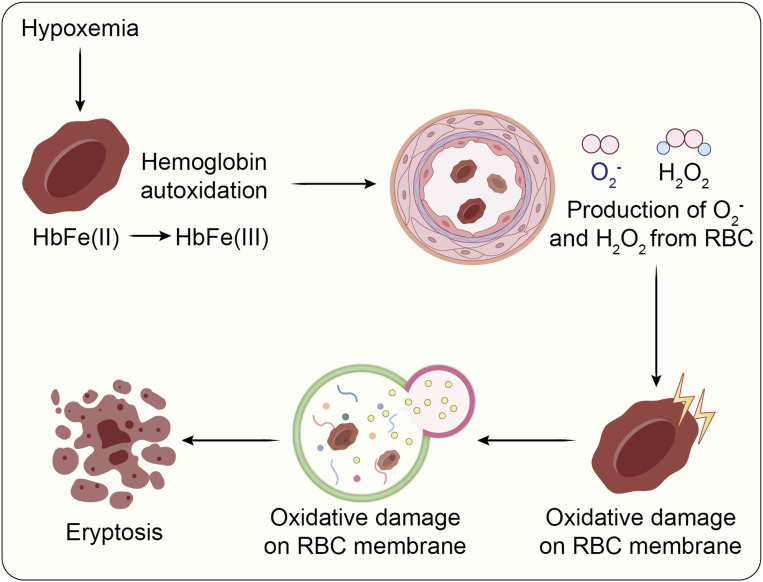
Under low oxygen conditions, hemoglobin undergoes autoxidation, producing superoxide anions (O_2_^−^) and hydrogen peroxide (H_2_O_2_). In CKD, a compromised antioxidant defense system is inadequate to counteract oxidative injury. The resulting aggravation of oxidative stress promotes the onset of eryptosis. Redrawn from Dias et al. (2020), licensed under CC BY.

As the key transporters of oxygen in the body RBCs are continually exposed to reactive oxygen species (ROS). A disturbance in the balance between pro-oxidants and antioxidants leads to oxidative stress, which inflicts molecular harm through free radicals and redox-active compounds (Sies, 2020; Sies, 2015). Under physiological conditions, redox homeostasis is maintained via strict control over ROS generation and elimination (Tsutsui et al., 2011). However, when antioxidative systems become saturated, RBCs accumulate oxidative lesions, provoking biochemical alterations and advancing pathological developments (Bardyn et al., 2018). An important origin of ROS within erythrocytes is the autoxidation of hemoglobin, generating superoxide radicals (Cimen, 2008). Under low oxygen tension, RBCs upregulate superoxide formation, which dismutates into hydrogen peroxide. This H_2_O_2_ can diffuse out of erythrocytes and provoke inflammatory activation in the lung’s microvascular endothelium (Kiefmann et al., 2008). Given the abundance of hemoglobin in blood, even slight elevations in autoxidation can disrupt redox balance—particularly in patients with compromised antioxidant responses, such as those with CKD. Furthermore, ROS derived from H_2_O_2_ and hydroxyl radicals inflict damage on erythrocyte membrane proteins and lipids, resulting in reorganization of the cytoskeleton, loss of membrane stability and deformability, and eventually eryptosis (Sudnitsyna et al., 2020). As shown in Figure 4, without mitochondria or lysosomes, RBCs possess no self-repair mechanisms and must rely entirely on enzymatic and non-enzymatic antioxidant systems. Central elements of these systems comprise superoxide dismutase, catalase, glutathione peroxidase, peroxiredoxin 2, and related biochemical pathways (Gonzales et al., 1984; Lee et al., 2003). In CKD, and especially at the end-stage renal disease (ESRD) phase, oxidative stress becomes profoundly intensified. Studies report an inverse relationship between oxidative stress indicators and both glomerular filtration rate and dialysis duration (Terawaki et al., 2004). ESRD patients exhibit lowered activity of glutathione peroxidase and elevated glutathione reductase levels (Ceballos-Picot et al., 1996; Roxborough et al., 1999). Hemodialysis procedures further amplify oxidative damage via membrane interactions and loss of antioxidants (Dae et al., 2019). Oxidative stress also impairs erythrocyte membrane integrity and permeability, elevating cellular fragility. These effects are mediated through activation of calcium channels, externalization of PS, accumulation of ceramide, emission of microvesicles, and decreased expression of band 3 protein (Dae et al., 2019; Bissinger et al., 2019).

While oxidative stress represents a fundamental mechanism in red blood cell (RBC) senescence, it is not the exclusive driver of this process. CD47, an integral membrane receptor, serves as an inhibitory signal that mitigates phagocytosis by binding to the macrophage receptor SIRPα. In contrast, phosphatidylserine (PS) exposure on the RBC surface functions as a pro-phagocytic (“eat me“) signal, promoting recognition and clearance by macrophages (Lutz and Bogdanova, 2013; Arias et al., 2017). Furthermore, Burger et al. demonstrated that oxidative stress-induced aging elicits a conformational shift in CD47, facilitating its interaction with thrombospondin-1 and thereby converting it into a phagocytosis-promoting signal (Burger et al., 2012). The influence of oxygen tension is underscored by observations that RBCs from males undergoing physical training under hypoxic conditions (15% O_2_) exhibited decreased CD47 expression, along with reduced levels of cytoskeletal proteins such as actin and spectrin. Hypoxia also impaired RBC deformability—a critical determinant of physiological function—through activation of the Gardos channel (Mao et al., 2011). Additionally, diminished CD47 expression has been reported in RBCs from individuals with chronic CKD (Antonelou et al., 2011). The intricate mechanisms governing RBC senescence continue to be an active area of investigation.

### 3.3 Inflammation

In CKD patients, the accumulation of uremic toxins and chronic immune activation drive the overproduction of pro-inflammatory cytokines, including C-reactive protein (CRP), interleukin-6 (IL-6), and interleukin-1β (IL-1β) (Coyne et al., 2017; David et al., 2016). These cytokines not only disrupt systemic iron homeostasis but also directly target mature erythrocytes to accelerate their apoptotic death (Babitt and Lin, 2012; Ogawa et al., 2018). *In vitro* studies have elucidated a key mechanistic pathway underlying this effect: high concentrations of IL-6 and IL-1β excessively activate Ca^2+^ channels on the erythrocyte membrane, leading to a pathological increase in intracellular Ca^2+^ influx. This dysregulated Ca^2+^ overload disrupts the structural integrity of the erythrocyte membrane—including damage to cytoskeletal proteins and lipid peroxidation—and triggers downstream apoptotic signaling cascades (Wang et al., 2022). Consistent with these findings, clinical observations in CKD cohorts have demonstrated a positive correlation between serum levels of IL-6/IL-1β and markers of erythrocyte membrane damage (e.g., increased osmotic fragility), as well as an inverse correlation with eryptosis (Mehta et al., 2017). A critical consequence of inflammation-induced erythrocyte membrane damage is the externalization of PS—a phospholipid normally confined to the inner leaflet of the erythrocyte membrane. In healthy RBCs, PS asymmetry is maintained by ATP-dependent flippases; However, Ca^2+^ overload (induced by pro-inflammatory cytokines) inhibits these flippases and activates scramblases, resulting in PS exposure on the cell surface (Lanser et al., 2021). The pro-apoptotic influence of inflammation on erythrocytes is mutually reinforced by uremic conditions in CKD. Uremic solutes, including indoxyl sulfate and p-cresyl sulfate, not only stimulate cytokine release via toll-like receptor 4 (TLR4) activation in immune cells but also prime erythrocytes for cytokine-derived Ca^2+^ influx by weakening antioxidative mechanisms (e.g., depleting glutathione) (Dinkla et al., 2016; Schuettpelz et al., 2014; Ganz, 2019). This interaction establishes a self-perpetuating cycle: uremia-induced inflammation hastens erythrocyte apoptosis, and apoptotic cell materials further stimulate immune activation and additional cytokine secretion (Paulson et al., 2020). Moreover, inflammation-induced elevation of fibroblast growth factor 23 (FGF23)—often heightened in CKD—facilitates erythrocyte apoptosis by suppressing EPO response and raising intracellular ROS levels (Czaya and Faul, 2019; Coe et al., 2014). ROS accumulation mediated by FGF23 worsens Ca^2+^ influx and PS exposure, thereby promoting faster erythrocyte removal (Mehta et al., 2017).

### 3.4 Iron

While iron deficiency (ID) has long been recognized as a driver of ineffective erythropoiesis in CKD, emerging evidence highlights that both iron deficiency and iron overload disrupt RBC longevity by promoting eryptosis—the programmed, non-hemolytic death of mature erythrocytes characterized by PS externalization, cell shrinkage, and cytoskeletal rearrangement (Lang K. S. et al., 2005; Lang F. et al., 2017). Iron is indispensable for erythrocyte function, serving as the central cofactor in hemoglobin and a critical regulator of redox balance (Kempe et al., 2006; Altamura et al., 2020). In CKD, iron homeostasis is perturbed by dual insults: reduced intestinal iron absorption (driven by hepcidin-mediated ferroportin degradation (Battaglia et al., 2020)) and uremic inflammation, which amplifies iron sequestration in macrophages (Cooke et al., 2013; Sasu et al., 2010). This dysregulation creates a paradox: iron deficiency limits hemoglobin synthesis, while iron overload induces oxidative stress—both converging to accelerate eryptosis and shorten RBC lifespan. Iron deficiency enhances eryptosis primarily through oxidative stress amplification and calcium (Ca^2+^) dyshomeostasis. Iron is a key cofactor for glutathione peroxidase 4 (GPX4), a lipid repair enzyme that neutralizes membrane lipid hydroperoxides to maintain RBC integrity (Liang et al., 2023). In iron-deficient states, GPX4 activity declines, leading to reactive oxygen species (ROS) accumulation and lipid peroxidation—potent triggers of eryptosis (Rochette et al., 2022). *In vivo* studies in iron-deficient mice confirm this: erythrocytes exhibit increased PS exposure, reduced glutathione (GSH) levels, and accelerated clearance, with these defects reversed by iron repletion (Kempe et al., 2006). Iron deficiency also disrupts Ca^2+^ homeostasis: non-selective cation channels in the RBC membrane—critical for Ca^2+^ entry—are upregulated by iron depletion, possibly via ROS-mediated gating. Increased intracellular Ca^2+^ activates Ca^2+^-dependent K^+^ channels (Gardos channels), promoting K^+^ efflux and cell shrinkage, while also stimulating scramblases that drive PS externalization (Lang et al., 2003). *In vitro*, iron chelation with deferoxamine mimics these effects, inducing dose-dependent eryptosis in human erythrocytes, confirming iron depletion *per se* primes RBCs for apoptotic death (Kempe et al., 2006). Conversely, iron overload—often secondary to repeated iron supplementation or transfusions in CKD—exacerbates eryptosis by fueling lipid peroxidation and membrane damage. Excess iron increases the labile iron pool (LIP) in erythrocytes, a redox-active fraction that catalyzes ROS generation via the Fenton reaction. This ROS surge induces peroxidative damage to membrane lipids and proteins (e.g., band 3, a key cytoskeletal protein), disrupting phospholipid asymmetry and triggering PS externalization (Turpin et al., 2021; Low et al., 1985; Bratosin et al., 2001). In hemochromatosis (a model of systemic iron overload), erythrocytes display increased PS exposure, elevated calpain activity (a cysteine protease mediating cytoskeletal degradation during eryptosis), and reduced deformability—changes that enhance splenic macrophage clearance (du Plooy et al., 2018; Matarrese et al., 2005). Similarly, in CKD patients with transfusional iron overload, erythrocytes show increased osmotic fragility and higher eryptosis rates, correlating with serum non-transferrin-bound iron (NTBI) levels (Matarrese et al., 2005). NTBI directly binds the RBC membrane, worsening peroxidative damage and sensitizing erythrocytes to eryptosis (Patel and Ramavataram, 2012). Iron overload also intersects with ferroptosis—a iron-dependent, lipid peroxidation-driven cell death pathway in nucleated cells—to amplify eryptosis (Li et al., 2024). Though erythrocytes lack mitochondria (a central ferroptotic player), they share key mediators: GPX4 and GSH (Altamura et al., 2020; Battaglia et al., 2020). Excess iron inhibits GPX4 in erythrocytes, mirroring ferroptosis and leading to unchecked lipid peroxidation and eryptosis (Yang et al., 2014). In CKD, uremic toxins (e.g., indoxyl sulfate) synergize with iron overload to suppress GPX4, further enhancing ROS-mediated eryptosis (Dias et al., 2021).

## 4 The role of eryptosis in patients

### 4.1 The role of eryptosis in non-dialysis-dependent patients

**FIGURE 5:**
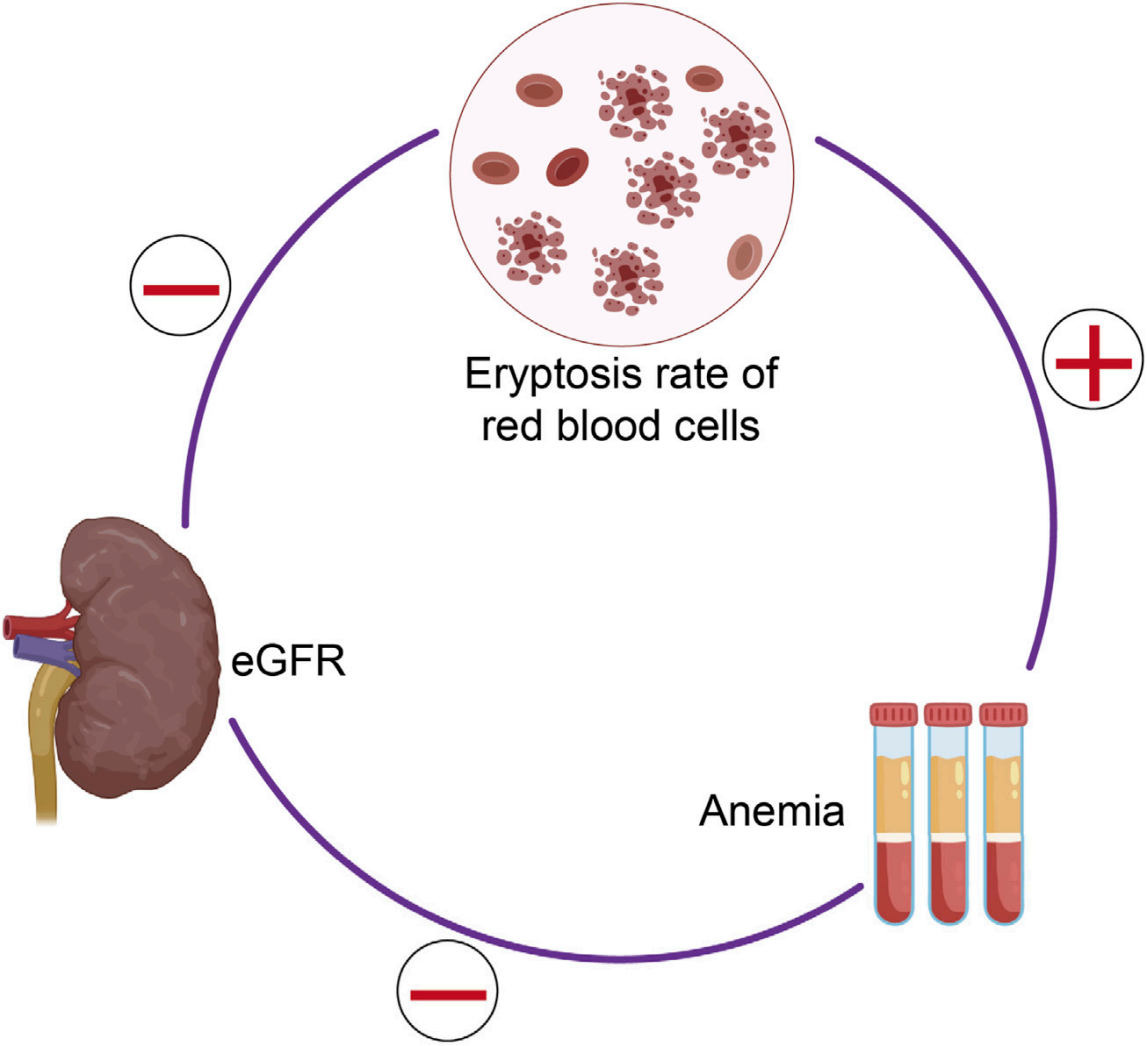
This schematic representation depicts the complex interplay among eryptosis, estimated glomerular filtration rate (eGFR), and anemia. The diagram highlights that the rate of eryptosis exhibits an inverse correlation with eGFR and a direct association with anemia severity. Furthermore, eGFR itself is inversely related to the presence and extent of anemia. Collectively, these correlations indicate that heightened eryptosis is closely linked to the progression of chronic kidney disease and the development of associated anemia. Redrawn from Dias et al. (2020), licensed under CC BY.

Eryptosis, the programmed death of erythrocytes characterized by PS exposure on the cell membrane, has emerged as a critical contributor to renal anemia in non-dialysis-dependent chronic kidney disease (NDD-CKD), with estimated glomerular filtration rate (eGFR) identified as its key determinant (Bissinger et al., 2024). Bissinger et al. conducted a cross-sectional study involving 122 NDD-CKD patients (spanning stages G1 to G5) and 133 healthy controls, demonstrating that NDD-CKD patients exhibited a ∼1.4-fold higher eryptosis rate (quantified via annexin V binding using flow cytometry) compared to controls. This elevated eryptosis was tightly linked to progressive eGFR decline: eryptosis levels were significantly higher in patients with CKD stages G3b and above relative to controls, and a strong negative correlation was observed between eryptosis rate and eGFR across the entire cohort (r = −0.45, P < 0.001). Notably, eryptosis correlated with GFR stages but not with albuminuria stages, and multiple linear regression analysis confirmed eGFR as the sole independent predictor of eryptosis in both the full cohort (*r* ^2^ = 0.22) and the NDD-CKD subgroup (*r* ^2^ = 0.10). The clinical relevance of enhanced eryptosis in NDD-CKD lies in its association with renal anemia, a major complication of progressive kidney disease. NDD-CKD patients in this study had significantly lower hemoglobin (Hb) concentrations (12.4 [11.1–13.7] g/dL) than healthy controls (13.8 [13.0–14.8] g/dL, P < 0.001), and eryptosis exhibited a negative correlation with Hb levels (r = −0.33, P < 0.001). This link between eryptosis and anemia is particularly impactful given the inadequate erythropoietic compensation in NDD-CKD: despite reduced RBC survival, NDD-CKD patients showed only a modest increase in reticulocyte count and an insufficient reticulocyte production index (RPI <2), accompanied by unelevated plasma erythropoietin (EPO) concentrations. Such impaired erythropoiesis, combined with accelerated eryptosis, creates a “double hit“ that exacerbates anemia as CKD progresses. Mechanistically, ceramide—a key mediator of calcium-independent eryptosis—was found to be significantly elevated in NDD-CKD patients (15.5 [14.0–16.6] vs. 15.0 [14.9–15.1] in controls, P = 0.002), whereas intracellular calcium and reactive oxygen species (ROS) showed no statistically significant differences (though ROS trended higher). Notably, *in vitro* incubation of healthy RBCs with plasma from NDD-CKD patients did not induce higher eryptosis compared to control plasma, a finding attributed to relatively lower uremic toxin concentrations in NDD-CKD (vs. dialysis-dependent CKD) due to residual renal function. This contrasts with observations in dialysis patients, where uremic plasma components (e.g., indoxyl sulfate, p-cresyl sulfate) strongly trigger eryptosis, highlighting a modality-specific difference in eryptosis drivers (Bissinger et al., 2025). Collectively, these findings establish eryptosis as a pathophysiologically relevant process in NDD-CKD, where eGFR decline drives enhanced RBC death that contributes to anemia development, which was illustrated in Figure 5. Targeting eryptosis—alongside optimizing EPO and iron therapy—may represent a novel strategy to improve anemia management in NDD-CKD, though further longitudinal studies are needed to confirm causality and identify actionable molecular targets.

### 4.2 Eryptosis in hemodialysis (HD) patients

Patients undergoing HD exhibit significantly higher eryptosis levels compared to healthy individuals, underscoring the profound impact of ESRD on erythrocyte survival (Abed et al., 2014). Multiple studies have attempted to characterize eryptosis dynamics before and after HD sessions, but inconsistencies persist across findings (Meyring-Wösten et al., 2017; Caprara et al., 2024). These discrepancies are primarily attributed to methodological limitations, including small sample sizes (insufficient statistical power to detect clear trends) and the analysis of only single or a limited number of HD sessions—factors that fail to capture the long-term effects of HD on eryptosis.

Key mediators of eryptosis in HD patients include uremic toxins, oxidative stress, and parathyroid hormone (PTH). Hefny et al. investigated 85 patients with stage 5 dialysis-dependent CKD (CKD5d) and demonstrated via linear regression analysis that PTH levels are independently associated with eryptosis, that was assessed by flow cytometry. This finding suggests PTH may represent a novel pathogenic link between hyperparathyroidism and renal anemia in HD patients, as elevated PTH exacerbates erythrocyte death (Hefny et al., 2022). Bissinger et al. further confirmed elevated eryptosis in HD patients and reported positive correlations between eryptotic cell percentages and levels of reactive oxygen species (ROS) and ceramide—two key drivers of oxidative stress and cell apoptosis (Bissinger et al., 2016). Additionally, they observed associations between PS-exposing erythrocytes (a hallmark of eryptosis) and both erythropoietin (EPO) dosage and reticulocyte percentages, highlighting the interplay between eryptosis and erythropoietic compensation (Bissinger et al., 2016). Beyond conventional HD, combination therapies have been evaluated for their effects on eryptosis and biocompatibility. Marcello et al. conducted a preliminary observational study of 7 chronic HD patients to assess hemodialysis combined with hemadsorption (HA + HD) using the HA130 cartridge. They found no significant differences in eryptosis levels before and after HA + HD treatment, while confirming efficient removal of middle-molecular-weight uremic toxins and protein-bound uremic toxins (PBUTs) without compromising biocompatibility. This suggests HA + HD may offer a therapeutic option to mitigate uremic toxicity without exacerbating erythrocyte death (Marcello, 2024). EPO, a cornerstone of anemia management in ESRD, exerts cytoprotective effects on mature erythrocytes by attenuating calcium influx, ROS generation, and caspase activation—ultimately reducing eryptosis. However, a subset of ESRD patients develops EPO resistance, often linked to chronic inflammation, iron dysregulation, and uremic toxin accumulation. These factors impair EPO receptor signaling, diminishing its protective effects and potentially exacerbating eryptosis. Understanding EPO responsiveness and its interaction with erythrocyte homeostasis is therefore critical for optimizing anemia management in HD patients (Bissinger et al., 2016). Meyring et al. further explored eryptosis in HD patients by investigating its relationship with erythrocyte sodium sensitivity. Compared to healthy controls, HD patients exhibited higher erythrocyte sodium sensitivity before treatment (which remained unchanged during HD), while eryptosis decreased post-treatment (Meyring-Wösten et al., 2017).

### 4.3 Eryptosis in peritoneal dialysis (PD) patients

Similar to HD, PD patients also experience dysregulated eryptosis, though key differences in underlying mechanisms and clinical correlates have been observed. Bissinger et al. reported higher eryptosis levels in PD patients compared to HD patients, with a positive correlation between eryptosis and dialysate volume. They hypothesized that glucose-based dialysate components may stimulate eryptosis, suggesting dialysate composition plays a role in erythrocyte damage (Bissinger et al., 2016). In contrast, Vos et al. used chromium-51 labeling to assess RBC survival in 14 HD and 5 PD patients and found no significant differences between groups (Vos et al., 2011). This discrepancy may stem from small sample sizes (particularly for PD patients) and variations in laboratory techniques, emphasizing the need for larger, standardized studies. Virzì et al. conducted a comprehensive analysis of 46 stable PD patients and confirmed that eryptosis rates are significantly higher in PD patients than in healthy controls. Notably, common PD comorbidities (diabetes mellitus, arterial hypertension, cardiovascular disease) and key PD parameters (continuous ambulatory vs. automated PD, Kt/Vurea values ≤ 1.7 vs. >1.7, history of peritonitis) did not influence eryptosis. Instead, eryptosis was negatively associated with residual renal function: PD patients with weekly creatinine clearance ≥45 L/week/1.73 m^2^ or residual diuresis (n = 23) exhibited lower eryptosis levels. In the subset with residual diuresis, eryptosis percentages correlated negatively with residual glomerular filtration rate (rGFR) and diuresis volume, suggesting progressive loss of residual renal function (and consequent reduced uremic toxin clearance) drives eryptosis elevation (Virzì et al., 2019).

Inflammation is a key driver of eryptosis in PD patients, particularly during PD-related peritonitis—a common and severe complication. Virzì et al. compared eryptosis and inflammatory markers (CRP, IL-6, IL-1β) in 31 PD patients with acute peritonitis and 34 control PD patients (no recent inflammation/peritonitis). On the first day of peritonitis, eryptosis was threefold higher in the peritonitis group, with strong positive correlations between eryptosis and all inflammatory indices (Virzì et al., 2022). This was supported by Bester et al., who demonstrated that IL-1β, IL-6, and IL-8 (proinflammatory cytokines) promote whole-blood hypercoagulability and alter erythrocyte membrane structure; IL-8, in particular, induced visible membrane changes and initiated eryptosis (Bester and Pretorius, 2016). C-reactive protein (CRP), a classic inflammatory marker, has also been identified as a trigger for eryptosis. Strong associations between CRP levels and eryptosis have been confirmed in acute inflammatory conditions (e.g., peritonitis, acute appendicitis) (Virzì et al., 2022; Abed et al., 2017). *In vitro* studies further validated these findings, showing that plasma from inflamed patients (e.g., PD patients with peritonitis) induces eryptosis in healthy donor RBCs. Virzì et al. extended these observations by linking systemic eryptosis to local peritoneal inflammation in PD patients. They found significant positive correlations between eryptosis levels and peritoneal biomarkers (peritoneal white blood cells [pWBC], peritoneal neutrophil gelatinase-associated lipocalin [pNGAL], IL-6, IL-1β) in PD effluent (PDE) (Virzì et al., 2024). This highlights a potential connection between systemic eryptosis and local peritoneal inflammation, while confirming that eryptosis is primarily influenced by blood composition—especially in inflammatory states.

## 5 Current therapeutic strategies targeting eryptosis in renal anemia

To date, the management of renal anemia in chronic kidney disease (CKD) has primarily focused on correcting erythropoietin (EPO) deficiency and iron dysregulation; However, emerging evidence from preclinical and clinical studies highlights that many of these standard therapies also exert beneficial effects by modulating eryptosis—an often-overlooked contributor to shortened RBC lifespan in CKD. This section synthesizes the mechanisms by which current therapeutic interventions (including EPO and its analogs, nitric oxide (NO) donors, and antioxidants) counteract accelerated eryptosis, thereby extending RBC survival and alleviating renal anemia, while also discussing their clinical utility, limitations, and relevance across CKD stages.

### 5.1 EPO

EPO is a classic regulator of erythropoiesis, but it also exhibits direct anti-eryptotic effects. EPO protects erythrocytes from oxidative stress-induced eryptosis by inhibiting Ca^2+^-permeable cation channels and reducing ROS generation (Sun et al., 2018). Additionally, EPO modulates the activity of anti-eryptotic kinases (e.g., AMP-activated protein kinase, AMPK) to maintain erythrocyte membrane integrity (Föller et al., 2009b; Myssina et al., 2003). The introduction of erythropoietin-stimulating agents (ESAs) in the 1980s represented a major therapeutic advance for renal anemia, significantly enhancing the quality of life in end-stage renal disease (ESRD) patients while reducing dependence on blood transfusions (Costa et al., 2008). ESA therapy has also been associated with decreased dialysis frequency and lower demand for healthcare resources, particularly among CKD patients requiring dialysis (Spinowitz et al., 2019). However, follow-up studies indicate that nearly 10% of individuals treated with ESAs develop resistance, a condition often linked to iron deficiency and systemic inflammation (van der Putten et al., 2008; Adamson, 2009). Dose escalation of ESAs has been correlated with elevated risks of malignancy, all-cause mortality, and hospitalization rates (René et al., 2017; Pérez-García et al., 2018). Although ESAs are widely used in CKD-related anemia, the comparative safety and mortality risks of different ESA types remain incompletely understood (Sakaguchi et al., 2019). Randomized controlled trials, including CHOIR, CREATE, and the Normal Hematocrit Trial, demonstrated that targeting normal hemoglobin levels in CKD or hemodialysis patients could lead to adverse outcomes, prompting a shift toward lower Hb targets and increased use of RBC transfusions (Szczech et al., 2008; Besarab et al., 1998; Eckardt et al., 2009).

### 5.2 Nitric oxide (NO) and NO donors

NO is a suppressor of eryptosis, independent of Ca^2+^ regulation. Even eryptosis induced by the calcium ionophore ionomycin is attenuated by NO donors (e.g., sodium nitroprusside) (Nicolay et al., 2008). NO exerts its anti-eryptotic effect by activating soluble guanylate cyclase (sGC), leading to cyclic guanosine monophosphate (cGMP) accumulation and subsequent activation of cGMP-dependent protein kinase I (cGKI). cGKI inhibits the activation of scramblase (responsible for PS externalization) and stabilizes erythrocyte membrane phospholipid asymmetry (Föller et al., 2008a; Montfort et al., 2017). Role in Renal Anemia In renal disease, endothelial NO synthase (eNOS) activity is reduced due to uremic toxins and oxidative stress, leading to decreased NO bioavailability (Owusu et al., 2012). Mice deficient in eNOS exhibit faster erythrocyte clearance and increased PS externalization, consistent with enhanced eryptosis. Supplementation with NO donors (e.g., sodium nitroprusside) restores NO-cGMP-cGKI signaling in erythrocytes, inhibits PS externalization, and prolongs erythrocyte lifespan in CKD models (Föller et al., 2008a). In end-stage renal disease (ESRD). Patients HD, NO donors reduce the percentage of eryptotic erythrocytes and improve anemia parameters by decreasing erythrocyte clearance (Abed et al., 2014).

### 5.3 Antioxidants

Antioxidants represent a critical class of eryptosis inhibitors, primarily targeting oxidative stress—a central driver of accelerated eryptosis in chronic kidney disease (CKD)-related renal anemia. In CKD, uremic toxin accumulation (e.g., indoxyl sulfate, acrolein), impaired antioxidant defense systems, and persistent inflammation collectively promote excessive production of reactive oxygen species (ROS), which disrupt erythrocyte membrane integrity, activate Ca^2+^-permeable cation channels, and trigger downstream eryptotic events (e.g., phosphatidylserine (PS) externalization, cell shrinkage, and cytoskeleton degradation) (Bissinger et al., 2019). To systematically summarize the diverse antioxidant inhibitors of eryptosis and their therapeutic relevance in renal anemia, Table 2 provides a detailed overview of their mechanisms, effects, and supporting evidence. N-acetyl-L-cysteine (NAC), a well-characterized thiol antioxidant and precursor of reduced glutathione (GSH), not only scavenges intracellular ROS directly but also replenishes depleted GSH stores in erythrocytes, thereby inhibiting ROS-mediated Ca^2+^ influx and PS externalization induced by eryptosis triggers such as desipramine or uremic toxins; *in vivo* studies further confirm NAC prolongs the half-life of circulating erythrocytes by interfering with efferocytosis of eryptotic cells, directly contributing to improved erythrocyte survival in CKD (Vota et al., 2012; Pan et al., 2023). Vitamin E, a lipid-soluble antioxidant, localizes to erythrocyte membranes, scavenges lipid peroxyl radicals, and maintains membrane fluidity and integrity, which mitigates ROS-induced membrane damage and Ca^2+^ permeation; clinical studies in CKD patients show vitamin E supplementation reduces erythrocyte lipid peroxidation and enhances deformability, complementing EPO therapy to alleviate renal anemia (Gökkuşu and Mostafazadeh, 2003; Kobayashi et al., 2003; Galli et al., 2022). Resveratrol, a natural polyphenol, exerts anti-eryptotic effects by suppressing ROS-dependent signaling pathways that regulate Ca^2+^ mobilization and PS externalization, while also reducing inflammation-associated oxidative stress in CKD, thus decreasing erythrocyte susceptibility to eryptosis (Qadri et al., 2009). Other antioxidants, including salidroside which protects erythrocytes against H_2_O_2_-induced eryptosis by enhancing antioxidant enzyme activity (Qian et al., 2012), Trolox (a water-soluble vitamin E analog that inhibits ROS-driven Ca^2+^ entry and eryptosis (Celik et al., 2024), and GSH itself which directly neutralizes ROS and maintains redox homeostasis in erythrocytes (Georgiou-Siafis and Tsiftsoglou, 2023), further support the role of antioxidant-based strategies in suppressing eryptosis. Collectively, these antioxidants alleviate renal anemia by reducing premature erythrocyte death, prolonging erythrocyte lifespan in circulation, and addressing the oxidative stress imbalance inherent to CKD, making them promising adjuvant therapeutic agents alongside conventional EPO or iron supplementation.

**TABLE 2:** Core antioxidant inhibitors of eryptosis in renal anemia.

Antioxidant type	Core mechanism of eryptosis inhibition	Key role in renal anemia	References
N-Acetyl-L-Cysteine (NAC)	Scavenges intracellular ROS; Replenishes depleted GSH; Inhibits ROS-mediated Ca^2+^ influx/PS externalization; Blocks efferocytosis of eryptotic cells	Prolongs the half-life of circulating erythrocytes Improves erythrocyte survival in CKD	Vota et al. (2012), Pan et al. (2023)
Vitamin E	Scavenges lipid peroxyl radicals (membrane-localized); Maintains membrane integrity; Mitigates ROS-induced Ca^2+^ permeation.	Reduces erythrocyte lipid peroxidation; Enhances deformability; Complements EPO.	Gökkuşu and Mostafazadeh (2003), Kobayashi et al. (2003), Galli et al. (2022)
Resveratrol	Suppresses ROS-dependent Ca^2+^/PS signaling; Reduces inflammation-associated oxidative stress.	Decreases erythrocyte eryptosis susceptibility; Prolongs lifespan in CKD.	Qadri et al. (2009)
Salidroside	Enhances antioxidant enzyme activity	Reduces oxidative stress-driven premature erythrocyte death.	Qian et al. (2012)
Trolox	Inhibits ROS-driven Ca^2+^ entry into erythrocytes.	Alleviates oxidative stress-induced eryptosis.	Celik et al. (2024)
Reduced Glutathione (GSH)	Directly neutralizes ROS; Maintains erythrocyte redox homeostasi	Mitigates oxidative stress-induced premature death.	Georgiou-Siafis and Tsiftsoglou (2023)

## 6 Future directions and clinical perspectives

Despite significant advances in delineating the role of eryptosis in the pathogenesis of renal anemia—including its regulation by uremic toxins (e.g., indoxyl sulfate, acrolein, oxidative stress, and inflammatory mediators—critical gaps remain in translating mechanistic insights into clinical practice, presenting several promising avenues for future research. First, eryptosis holds substantial potential as a novel biomarker for risk stratification and therapeutic monitoring in CKD patients. Integrating markers of eryptosis (Table 3) into routine clinical monitoring could enable early identification of patients at high risk of progressive anemia or inflammation-driven erythrocyte loss, particularly in non-dialysis-dependent CKD (NDD-CKD) and peritoneal dialysis (PD) patients with residual renal function. Such monitoring may also help differentiate anemia etiologies and guide personalized therapy adjustments, as demonstrated by the link between eryptosis and erythropoietin resistance in hemodialysis (HD) patient. Second, targeting eryptosis pathways offers new therapeutic opportunities: strategies to mitigate eryptosis could include optimizing clearance of eryptosis-inducing uremic toxins via advanced dialysis modalities (e.g., hemodialysis combined with hemadsorption [HA + HD] for protein-bound uremic toxins) or novel pharmacotherapies, such as inhibitors of Organic Anion Transporter 2 (OAT2) to block indoxyl sulfate uptake by erythrocytes, antioxidants (e.g., N-acetyl-L-cysteine, vitamin E) to counteract oxidative stress-mediated eryptosis, or modulators of Ca^2+^ signaling (e.g., NO donors) to stabilize erythrocyte membrane asymmetry. Additionally, exploring synergies between existing therapies could enhance anemia correction while reducing reliance on high-dose erythropoiesis-stimulating agents (ESAs) and their associated risks. HIF-PHI, such as roxadustat and vadadustat, have revolutionized renal anemia management by stimulating endogenous erythropoietin (EPO) and improving iron utilization. Beyond their erythropoietic effects, HIF-PHI may indirectly reduce apoptosis by mitigating oxidative stress and inflammation (Haase et al., 2024). Future studies should investigate whether HIF stabilization directly modulates erythrocyte apoptotic pathways, such as by upregulating anti-apoptotic genes or downregulating pro-apoptotic factors. While no clinical trial has tested this combination, the anti-apoptotic and antioxidant effects of HIF-PHIs provide a strong mechanistic rationale for synergistic use with agents targeting erythrocyte apoptosis pathways (e.g., Bcl-xL upregulation or NIX inhibition). In non-dialysis CKD (NDD-CKD) patients, roxadustat improved hemoglobin levels even in the presence of inflammation, suggesting enhanced erythrocyte survival under stress conditions (Zhang et al., 2021; Hirota, 2021). Third, further research is needed to optimize dialysis modality-specific effects on eryptosis: while PD patients exhibit higher eryptosis levels than HD patients, potentially due to glucose-based dialysate components, conflicting data on RBC survival across modalities highlight the need for large-scale, longitudinal studies to compare the impact of conventional HD, hemodiafiltration (HDF), and biocompatible PD solutions on eryptosis rates and long-term anemia outcomes. Finally, validating the prognostic value of eryptosis in prospective cohorts—building on preliminary findings linking eryptosis to cardiovascular risk and peritoneal inflammation severity—will be critical to establishing its clinical relevance beyond anemia management, potentially positioning eryptosis as a broader marker of uremic toxicity and systemic inflammation in CKD.

**TABLE 3:** A typical sign of eryptosis.

Detection category	Detection method	References
PS exposure	Fluorescein-conjugated annexin V by flow cytometry (FCM) (gold standard) or fluorescence microscopy; ELISA kits have also been developed	Aslam et al. (2024)
Aminophospholipid translocase	Reverses the orientation of externalized PS; activity is measured by a fatty acid-labeled phosphatidylserine (NBD-PS) fluorescent probe	Mandal et al. (2005)
Intracellular calcium	Fluo4/AM or X-Rhod-1, AM	Boulet et al. (2021)
Oxidative stress	DCF (H_2_DCFDA staining) Colorimetric or fluorogenic substrate-based methods (specific ROS detection)	Bissinger et al. (2015)
Cell shrinkage or swelling	FSC (gold standard) or MCV by an automated hematology analyzer	Larkin et al. (2024)
Senescence	β-galactosidase by fluorogenic probes	Bosmann (1971)

## 7 Conclusion

In summary, eryptosis has emerged as a pivotal mechanism contributing to the pathophysiology of renal anemia, particularly in the context of chronic kidney disease. While traditional paradigms have centered on erythropoietin deficiency and iron dysregulation, it is now evident that accelerated erythrocyte death—driven by uremic toxins, oxidative stress, chronic inflammation, and iron imbalance—plays a critical role in reducing red blood cell lifespan and exacerbating anemia. The complex interplay of these factors not only undermines the efficacy of conventional therapies such as ESAs and iron supplementation but also contributes to cardiovascular complications and poor clinical outcomes. Importantly, eryptosis represents a modifiable pathway, offering promising therapeutic targets. To realize this vision, future research maybe focus on standardizing the measurement of eryptosis in clinical settings, validating its role as a predictive biomarker, and conducting well-designed clinical trials to evaluate the efficacy and safety of novel anti-eryptotic agents. Embracing this integrated pathophysiological view is crucial for advancing towards more effective and personalized management strategies for patients with renal anemia.

## Abbreviations

AA, Arachidonic Acid; AE1, Anion Exchanger 1; AMPK, AMP-activated Protein Kinase; casp, Caspases; CDK4, Cyclin-dependent Kinase 4; C.E.R.A., Continuous Erythropoietin Receptor Activator; cGK, cGMP-dependent Protein Kinase; cGKI, cyclic GMP-dependent Protein Kinase type I; cGMP, cyclic Guanosine Monophosphate; CK1, Casein Kinase 1; CKD, Chronic Kidney Disease; COX, Cyclooxygenase; CRP, C-reactive Protein; eGFR, estimated Glomerular Filtration Rate; eNOS, endothelial Nitric Oxide Synthase; EPO, Erythropoietin; ESAs, Erythropoiesis-Stimulating Agents; ESRD, End-Stage Renal Disease; FGF23, Fibroblast Growth Factor 23; GSH, Glutathione; GPX4, Glutathione Peroxidase 4; Hb, Hemoglobin; HD, Hemodialysis; HDF, Hemodiafiltration; HIF-PHIs, Hypoxia-Inducible Factor Prolyl Hydroxylase Inhibitors; IAA, Indole-3-acetic Acid; ID, Iron Deficiency; IL-1β, Interleukin-1β; IL-6, Interleukin-6; IS, Indoxyl Sulfate; IV, Intravenous; JAK3, Janus Kinase 3; KCa3.1, Calcium-activated Potassium Channel 3.1; KCNN4, Potassium Calcium-activated Channel Subfamily N Member 4; KDIGO, Kidney Disease: Improving Global Outcomes; LIP, Labile Iron Pool; MAPK, Mitogen-activated Protein Kinase; MPs, Microparticles; MSK1/2, Mitogen- and Stress-activated Kinases 1/2; NAC, N-acetyl-L-cysteine; NDD-CKD, Non-Dialysis-Dependent Chronic Kidney Disease; NO, Nitric Oxide; NTBI, Non-Transferrin-Bound Iron; OAT1/3, Organic Anion Transporters 1/3; OAT2, Organic Anion Transporter 2; PAF, Platelet-activating Factor; PAK2, p21-activated Kinase 2; PBUTs, Protein-Bound Uremic Toxins; PD, Peritoneal Dialysis; PDE, PD Effluent; PGE2, Prostaglandin E2; p38 MAPK, p38 Mitogen-activated Protein Kinase; PKC, Protein Kinase C; PLA, Phospholipase A; PMCA, Plasma Membrane Ca^2+^ ATPase; PS, Phosphatidylserine; pNGAL, peritoneal Neutrophil Gelatinase-Associated Lipocalin; PTH, Parathyroid Hormone; pWBC, peritoneal White Blood Cells; RBC, Red Blood Cell; RBCL, Red Blood Cell Lifespan; ROS, Reactive Oxygen Species; RPI, Reticulocyte Production Index; SC, Subcutaneous; SCR, Scramblase; sGC, soluble Guanylate Cyclase; SM, Sphingomyelin; TLR4, Toll-like Receptor 4; TRPC, Transient Receptor Potential Canonical.
